# Supplementary material for: Immunogenicity and safety of a recombinant adenovirus type-5 COVID-19 vaccine in adults: Data from a randomised, double-blind, placebo-controlled, single-dose, phase 3 trial in Russia
Source: PLoS One. 2023 Mar 8;18(3):e0278878. doi: 10.1371/journal.pone.0278878 (PMC9994755; doi:10.1371/journal.pone.0278878)
Supplement: S1 Methods — (DOCX) [file pone.0278878.s003.docx]

**Statistical Methods**

Initially, as part of the interim analysis and to ensure 90% power for the between-group comparison of the primary variable, 180 participants were to be included. This was based on the assumptions of a 20% seroconversion rate in the Placebo group (based on quantitative analysis), advantage of the vaccine group of ≥30% (conservative assumption, odds ratio = 4), corrected two-sided significance level of 0.02616 (one-sided level of 0.01308) and the randomisation ratio of 3:1. Considering potential dropouts from the study during the initial observation period of 28 days (10% of participants), the total number of randomised participants was increased to 200. To provide more detailed safety and efficacy data (including age subgroups) and to descriptively present the frequency of confirmed COVID-19 cases that occurred within 6 months post-vaccination (except for COVID-19 cases that developed during the first 14 days after the vaccination), a sample size of 500 was selected.

For the cellular immune response analysis, a subset of 69 participants from the full analysis set (FAS) for immunogenicity analysis population were used. These were the participants that visited the Moscow clinic site, which was where the cellular immune response analysis was performed. In the phase 2 clinical study conducted in China, half as many participants were used in their cellular immune response analysis [1].

An unblinded interim analysis was originally planned and conducted in this clinical trial when obtaining partial information to evaluate the primary endpoint, gathered from the first 200 randomised volunteers and based on the results collected up to Day 28 (Visit 5), including the data for those who left the trial before Visit 5. Subsequently, an alpha spending function (that is, an increasing function of the proportion of the maximum sample size) of the Pocock type was employed to adjust the level of significance due to the multiple comparisons of the primary variable in the planned interim and final analysis sets. Analyses were performed with bilateral alpha levels of 0.02616 and 0.03039 on the interim and final analysis sets, respectively (with a total bilateral significance level of 5%).

Variables representing the seroconversion rate (the proportion of participants with at least a 4-fold increase in antibody titres) were tabulated by evaluation time‑points and treatment groups, and two-sided Clopper-Pearson 95% confidence intervals (CIs) presented. Comparative analysis of the primary endpoint (seroconversion, quantitative definition) was performed using the Cochran-Mantel-Haenszel test and the chi-squared test (or Fisher’s exact test).

Antibody GMTs and neutralising antibody GMTs were presented and compared by evaluation time points based on the calculated 95% CIs using two-way analysis of variance (ANOVA) following logarithmic transformation. Mean log-transformed differences between the study groups (vaccine and placebo) were evaluated with the corresponding 95% CI. The point estimates of the mean differences and the corresponding CIs were back-transformed.

Geometric mean fold-increases in antibody and neutralising antibody titres for each treatment group and each antibody GMT evaluation time point were assessed with the corresponding two-sided 95% CIs following log-transformation, point estimation of the difference and CIs, and back-transformation of obtained values. Between-group comparisons were performed using ANOVA following the logarithmic transformation. Results from the ELISpot assay to assess differences in cellular immunity were analysed using Mann-Whitney test with Bonferroni adjustment of p-values.

Statistical significance was accepted at p<0.05.

**REFERNCES**

[1] Zhu F, Guan X, Li Y, Huang J, Jiang T, Hou L, et al. Immunogenicity and safety of a recombinant adenovirus type-5-vectored COVID-19 vaccine in healthy adults aged 18 years or older: a randomised, double-blind, placebo-controlled, phase 2 trial. Lancet. 2020;396: 479–488. doi: 10.1016/S0140-6736(20)31605-6.
