# Supplementary material for: Immunogenicity and safety of a recombinant adenovirus type-5 COVID-19 vaccine in adults: Data from a randomised, double-blind, placebo-controlled, single-dose, phase 3 trial in Russia
Source: PLoS One. 2023 Mar 8;18(3):e0278878. doi: 10.1371/journal.pone.0278878 (PMC9994755; doi:10.1371/journal.pone.0278878)
Supplement: S1 Table — (DOCX) [file pone.0278878.s004.docx]

**S1 Table. Summary of Geometric Mean Titres and Seroconversion Rates Post-vaccination with Ad5-nCoV or Placebo (Full Analysis Set)**

| **Serum**  **Ab** | **Parameter** | **Ad5-nCoV**  **N=371** | | | | **Placebo**  **N=124** | | | |
| --- | --- | --- | --- | --- | --- | --- | --- | --- | --- |
|  |  | **Day 0** | **Day 14** | **Day 28** | **6 Month** | **Day 0** | **Day 14** | **Day 28** | **6 Month** |
| S protein | GMT (95% CI) | 50.3 (45.2,55.9) | 289 (259,321)^a^ | 677 (608,753)^a^ | 293 (263,327)^a^ | 50.3 (41.8,60.4) | 51.5 (42.7,62.2) | 61.3 (50.8,73.9) | 146 (119,179) |
|  | SC participants (n/N) | - | 239/359 | 329/363 | 222/353 | - | 1/118 | 8/119 | 41/101 |
|  | SCR, % (95% CI) | - | 66.6 (61.4,71.4) | 90.6 (87.2,93.4) | 62.9 (57.6,67.9) | - | 0.847 (0.022,4.63) | 6.72 (2.95,12.8) | 40.6 (30.9,50.8) |
| RBD | GMT (95% CI) | 50.1 (45.3,55.4) | 139 (125,154)^a^ | 405 (366,449)^a^ | 153 (138,170) | 50 (42.0,59.5) | 52.7 (44.1,63.0) | 58.2 (48.7,69.5) | 116 (95.2,140) |
|  | SC participants (n/N) | - | 152/359 | 285/363 | 140/353 | - | 2/118 | 7/119 | 35/101 |
|  | SCR, % (95% CI) | - | 42.3 (37.2,47.6) | 78.5 (73.9,82.6) | 39.7 (34.5,45.0) | - | 1.69 (0.206,5.99) | 5.88 (2.4,11.7) | 34.7 (25.5,44.8) |
| NAbs | GMT (95% CI) | 5 (4.55,5.5) | 8.78 (8,9.64)^a^ | 16.7 (15.3,18.3)^a^ | 19.5 (17.8,21.3)^a^ | 5 (4.25,5.89) | 5 (4.26,5.87) | 5.79 (4.96,6.76) | 10.3 (8.69,12.2) |
|  | SC participants (n/N) | - | 75/308 | 183/310 | 149/305 | - | 0/104 | 4/103 | 32/93 |
|  | SCR, % (95% CI) | - | 24.4 (19.7,29.5) | 59.0 (53.3,64.6) | 48.9 (43.1,54.6) | - | 0 (0,3.48) | 3.88 (1.07,9.65) | 34.4 (24.9,45.0) |
| Ad5 | GMT (95% CI) | 11.2 (9.49,13.3) | - | 36.5 (31,42.9)^a^ | 48.3 (41.1,56.8)^a^ | 8.83 (6.57,11.9) | - | 9.21 (6.96,12.2) | 15.7 (11.6,21.3) |
|  | SC participants (n/N) | - | - | 131/310 | 169/305 | - | - | 2/103 | 23/93 |
|  | SCR, % (95% CI) | - | - | 42.3 (36.7,48.0) | 55.4 (49.6,61.1) | - | - | 1.94 (0.236,6.84) | 24.7 (16.4,34.8) |

Ab, antibody; Ad5, adenovirus type 5; Ad5-nCoV, adenovirus type-5 vectored COVID-19 vaccine; ANOVA, analysis of variance; COVID-19, coronavirus disease 2019; GMT, geometric mean titre; N, total number of participants; n , number of participants who seroconverted; NAbs, neutralising antibodies against SARS-CoV-2; SARS-CoV-2, severe acute respiratory syndrome coronavirus 2; SC, seroconverted; SCR, seroconversion rate; RBD, receptor binding domain; S, spike

^a^ ANOVA, p<0.001

Note: The seroconversion rate was determined as the proportion of participants that seroconverted, defined by a four-fold increase in antibody titre compared to Day 0.
