# Supplementary material for: Immunogenicity and safety of a recombinant adenovirus type-5 COVID-19 vaccine in adults: Data from a randomised, double-blind, placebo-controlled, single-dose, phase 3 trial in Russia
Source: PLoS One. 2023 Mar 8;18(3):e0278878. doi: 10.1371/journal.pone.0278878 (PMC9994755; doi:10.1371/journal.pone.0278878)
Supplement: Attachment — Submitted filename: Plos One_Review.docx [file pone.0278878.s011.docx]

The authors present a study based on the phase 3 clinical data available for Ad5-nCoV in the European population. The study revolves about vaccinating a population with a single dose of Ad5-nCoV and others with placebo for the phase 3 trials. I have few suggestions so that the work presented here becomes more interesting for a broader audience.

I will strongly suggest revising the manuscript by compiling their data into a tabular form rather than writing similar sentences throughout the manuscript. Look at sentences: line number 325-358 and similar sentences.

In Abstract, line number 30: I guess that the authors are trying to say “seroconversion against the SARS-CoV-2 virus” not “neutralizing SARS-CoV-2 antibodies”.

Line 34 and 169: Authors should check if full length Spike protein was used in their study or the ectodomain of the spike protein. The spike protein of the SARS-CoV-2 is a type-I transmembrane protein; therefore, it would require the presence of detergents or other agents, which can keep them stable and soluble.

Line 151: It should be anti-SARS-CoV-2 virus not “anti-coronavirus”. Both have different meanings.

Line 543: Authors should provide a citation and try to reframe the sentence.

In the result section: I find that an explanation of their findings and observations are totally missing. They should clearly write: what is the take home message from every experiment. They have written observations but the scientific explanations are missing in their manuscript.

They should also clearly point out in their text, which figure they are discussing. For example, if Fig2 or Fig3 contains multiple figures then they should be marked as A, B, C, and D, and then each of them should be discussed in the text. Otherwise, it is difficult for a reader to grasp all the information.

Overall, the manuscript needs a major revision from authors so that it becomes more informative and interesting.
