# Supplementary material for: Immunogenicity and safety of a recombinant adenovirus type-5 COVID-19 vaccine in adults: Data from a randomised, double-blind, placebo-controlled, single-dose, phase 3 trial in Russia
Source: PLoS One. 2023 Mar 8;18(3):e0278878. doi: 10.1371/journal.pone.0278878 (PMC9994755; doi:10.1371/journal.pone.0278878)
Supplement: Attachment — Submitted filename: Comments.docx [file pone.0278878.s012.docx]

**Mohamed Ali**

**Comments:**

The present study is a multicenter, randomized, double-blind, placebo-controlled, phase 3 trial of a candidate vaccine developed in China and based on Ad5 vector. The study included 500 participants aged 18–85 years (mean age: 41.2 years), the immunogenicity, efficacy and safety of the Ad5-nCoV COVID-19 vaccine was assessed up to 6 months after vaccination. A single dose immunization with the Ad5- nCoV candidate vaccine led to a marked immune response which measured at day 14,28 and 6 months. Antibodies raised against SARS-CoV2 RBD, S and Ad5 antigens with variable ratios. Vaccination showed statistically significant immune response compared with the placebo unvaccinated participants. The study effort is highly appreciated.

We have some comments would that the authors could be reply

1. The sample size was relatively small.
2. The study needs more data about pre-existing immunity to Ad5 between the Russian people to differentiate the neutralizing antibodies raised against the target protein of the candidate SARS-CoV2 vaccine.
3. As a result of virus mutation, the study of immune response to Ad5 cassette for vaccine development is an urgent need.

Some typing corrections :

P.7. Raw 110,211 &229	change urinanalysis	to urine analysis

P.9,R.163	correct serums to sera

P. 10,R 166	change reproduction	to propagation

P.12, R.219	correct SARS-CoV3 to SARS-CoV2

P.15 paragraph “ Participants were…………………….pandemic” need more clarification

Fig 5 need to use contrasted colors to differentiate between lines
