# Supplementary material for: Immunogenicity and safety of a recombinant adenovirus type-5 COVID-19 vaccine in adults: Data from a randomised, double-blind, placebo-controlled, single-dose, phase 3 trial in Russia
Source: PLoS One. 2023 Mar 8;18(3):e0278878. doi: 10.1371/journal.pone.0278878 (PMC9994755; doi:10.1371/journal.pone.0278878)
Supplement: Attachment — Submitted filename: Response to Reviewers.DOCX [file pone.0278878.s013.DOCX]

Dear Editor Nagendra R. Hegde,

Thank you for giving us the opportunity to submit a revised manuscript, titled “*Immunogenicity and safety of a recombinant adenovirus type-5 COVID 19 vaccine in adults: data from a randomised, double-blind, placebo-controlled, single-dose, phase 3 trial in Russia*” for publication in *PLOS One*.

We appreciate the time and effort that you and the reviewers have dedicated to providing your valuable feedback and thoroughly reviewing this manuscript. We have addressed the remaining concerns from Reviewer 3. For your and the reviewers’ convenience, all changes are marked using track changes in the revised manuscript, that is, in the ‘Revised Manuscript with Track Changes.docx’ file.

Please find a point-by-point response to the reviewers’ comments and concerns below here. Please find the black comments followed by our reply in red.

Of note, upon review of the references cited in the manuscript, a few references have been updated or reported corrections. We have updated the following references as appropriate: Nussbaumer-Streit et al, 2020 (citation number 2 in the revised manuscript); Voysey et al, 2021 (citation number 4 in the revised manuscript); Logunov et al, 2020 (citaiton number 6 in the revised manuscript); and Logunov et al, 2021 (citation number 17 in the revised manuscript). The reference for Sui et al, 2004 (citation number 8 in the revised manuscript) was amended to include the corresponding doi. Additionally, review of Guidelines for Conducting Clinical Studies of Medicinal Products (citation number 14 in the revised manuscript) indicated that the incorrect webpage was provided. This reference has been corrected to the following in the revised manuscript: ’Mironov AN, editor. Guidelines for Preclinical Trials of Medicinal Products. Part 2. Moscow: Grif i K. 2012’.

We are looking forward to your response.

On behalf of the study authors,

Vitalina Dzutseva

# Reviewer 3

**Comment 1:** Line 354: the seroconversion rate and 95% CI for NAbs is not correct (what is written is for RBD).

Authors’ Response: Thank you for bringing this to our attention. We have corrected the text based on the information presented in Table S1. The updated text is located in Line 357 in the revised manuscript as well as part of the response to Comment 2 as presented below.

**Comment 2:** Lines 352-356: This sentence as worded is a little confusing, because the seroconversion rates are all from day 28 but the sentence talks about both day 14 and day 28.

Authors’ Response: We acknowledge that this initial phrasing was not clear. We have revised the sentence to emphasize that the data presented is from the seroconversion rates for Day 28. The revised text in the Results section is as follows (pg. 19, lines 352–357):

“For those vaccinated with Ad5-nCoV vs. the placebo, seroconversion rates for S protein, RBD, and NAbs were significantly elevated by Day 14 (p<0.001; Fig. 3A–3C and S1 Table), and the seroconversion rates peaked at Day 28 for S protein (90.6% [95% CI: 87.2, 93.4] vs. 6.72% [95% CI: 2.95, 12.8]), RBD (78.5% [95% CI: 73.9, 82.6] vs. 5.88% [95% CI: 2.4, 11.7]), and NAbs (59.0% [95% CI: 53.3, 64.6] vs. 3.88% [95% CI: 1.07, 9.65]) (p<0.001).”

**Comment 3:** Figure 3C (and related text and figure caption): The text says the comparison between the Ad4-nCoV group and placebo for NAbs was significant at 6 months (p<0.001), but the figure has a single * and the caption says * indicates p<0.05. Authors should either correct the figure or modify the sentence to match the results.

Authors’ Response: We acknowledge the oversight on our part as the p-value of p<0.001 corresponded to only the seroconversion rate for S protein after 6 months when comparing those in the Ad5-nCoV vs the Placebo group. We have clarified the text to clearly attribute the p-value with the corresponding data in the figure. The revised text in the Results section is as follows (pg. 19, lines 357–359):

“Seroconversion rates for S protein and NAbs in the Ad5-nCoV group were elevated after 6 months compared with the Placebo group (p<0.001 and p<0.05, respectively; Fig. 3A and 3C, and S1 Table), indicating that some protection remained at the later timepoint.”

**Comment 4:** line 377: "most participants low" should be "most participants had low"

Authors’ Response: We have revised the text as per your suggestion.

**Comment 5:** lines 379-380: authors added "Regardless of treatment group" to the start of the sentence, but the numbers seem to refer to the Ad5-nCoV group. Authors should clarify.

Authors’ Response: We apologize for the confusion. We have revised the text for clarity. The revised text in the Results section is as follows (pg. 20, lines 379–381):

“Of those in the Ad5-nCoV group, GMTs of anti-Ad5 participants with low pre-existing anti-Ad5 antibodies increased from 11.3 at baseline (n=317) to 36.5 at Day 28 (n=353), which then increased further to 48.3 (n=355) at Month 6.”

**Comment 6:** line 386: "based their" should be "based on their"

Authors’ Response: We have revised the text as per your suggestion.

**Comment 7:** line 443: authors report a correlation of -0.05 at 6 months, but the dot in Figure 6 appears to be above the dashed line at 0. Is the correlation negative?

Authors’ Response: We originally intended to use a “+” for this data point. This has been corrected in the revised text (pg. 24, line 444).

**Comment 8:** lines 444-447: I believe the authors want to refer to the magnitude of the correlation decreasing. Mathematically a negative number getting closer to 0 is actually increasing (but the magnitude would be the absolute value, so getting closer to 0 would be a decrease). Also, authors mention correlations decreasing from baseline to Month 6 for both S protein and nAbs, but there is not a correlation at baseline for nAbs in Figure 6.

Authors’ Response: We have revised the text for clarity to emphasize the correlation magnitudes. We also specify that the timeframe as the GMTs of NAbs were recorded between Day 14 and Month 6. The revised text in the Results section is as follows (pg. 23, lines 444–448):

“The correlation magnitudes of GMTs to S protein and neutralising SARS-CoV-2 antibodies decreased from Day 14 to Month 6 (Fig. 6). These changes in GMT correlations indicate that the relationship between levels of baseline Ad5 antibodies to the COVID-19 humoral immune response diminishes over time following vaccination.”

**Comment 9:** line 552. I believe "As listed in S5 Table" should be "As listed in S6 table"

Authors’ Response: This has been corrected as S6 Table specifically lists the injection reactions by severity.

**Comment 10:** lines 555-556: the percentages do not match those presented in S6 Table

Authors’ Response: The total percentages were reported instead of the percentages of those specifically in the Ad5-nCoV group. We have revised the text accordingly. The revised text in the Results section is as follows (pg. 28, lines 554–557):

“While moderate reactions rarely occurred in those that received the Ad5-nCoV vaccine, 15 participants (4%) in the Ad5-nCoV group experienced the following local post-vaccination reactions of moderate severity at the injection site: erythema (8 [2.2%]), pain (6 [1.6%]), swelling (5 [1.3%]), and induration (1 [0.3%]).”

**Comment 11:** line 559: it is confusing to see 152/365 and 38/75. I realize authors added a note under Tables 2 and 3 to clarify, but in the text, wouldn't it be easier (and less confusing) to report just the number of participants - readers can see the number of AEs in the table.

Authors’ Response: Thank you for this suggestion. We wanted to be as transparent as possible by reporting the numbers in the same format; however, this has created additional confusion. As per your suggestion we have reported only the number of participants. The revised text in the Results section is as follows (pg. 29, lines 560–562):

“As listed in Table 3, a total of 190 participants (38.3%) reported 440 AEs during the 6 months after vaccination, of which 152 (40.9%) participants from the Ad5-nCoV group reported 365 AEs and 38 (3.6%) participants from the Placebo group reported 75 AEs.”
